# Arsenic Trioxide Reduces Global Histone H4 Acetylation at Lysine 16 through Direct Binding to Histone Acetyltransferase hMOF in Human Cells

**DOI:** 10.1371/journal.pone.0141014

**Published:** 2015-10-16

**Authors:** Da Liu, Donglu Wu, Linhong Zhao, Yang Yang, Jian Ding, Liguo Dong, Lianghai Hu, Fei Wang, Xiaoming Zhao, Yong Cai, Jingji Jin

**Affiliations:** 1 School of Life Sciences, Jilin University, Changchun, Jilin 130012, China; 2 National Engineering Laboratory for AIDS Vaccine, Jilin University, Changchun City, Jilin 130012, China; 3 Key Laboratory for Molecular Enzymology and Engineering, the Ministry of Education, Jilin University, Changchun City, Jilin 130012, China; 4 Research Center for Drug Metabolism, School of Life Sciences, Jilin University, Changchun 130012, China; 5 School of Pharmacy, Changchun University of Traditional Chinese Medicine, Changchun 130117, China; The Walter and Eliza Hall of Medical Research, AUSTRALIA

## Abstract

Histone post-translational modification heritably regulates gene expression involved in most cellular biological processes. Experimental studies suggest that alteration of histone modifications affects gene expression by changing chromatin structure, causing various cellular responses to environmental influences. Arsenic (As), a naturally occurring element and environmental pollutant, is an established human carcinogen. Recently, increasing evidence suggests that As-mediated epigenetic mechanisms may be involved in its toxicity and carcinogenicity, but how this occurs is still unclear. Here we present evidence that suggests As-induced global histone H4K16 acetylation (H4K16ac) partly due to the direct physical interaction between As and histone acetyltransferase (HAT) hMOF (human male absent on first) protein, leading to the loss of hMOF HAT activity. Our data show that decreased global H4K16ac and increased deacetyltransferase HDAC4 expression occurred in arsenic trioxide (As_2_O_3_)-exposed HeLa or HEK293T cells. However, depletion of HDAC4 did not affect global H4K16ac, and it could not raise H4K16ac in cells exposed to As_2_O_3_, suggesting that HDAC4 might not directly be involved in histone H4K16 de-acetylation. Using As-immobilized agarose, we confirmed that As binds directly to hMOF, and that this interaction was competitively inhibited by free As_2_O_3_. Also, the direct interaction of As and C2CH zinc finger peptide was verified by MAIDI-TOF mass and UV absorption. In an *in vitro* HAT assay, As_2_O_3_ directly inhibited hMOF activity. hMOF over-expression not only increased resistance to As and caused less toxicity, but also effectively reversed reduced H4K16ac caused by As exposure. These data suggest a theoretical basis for elucidating the mechanism of As toxicity.

## Introduction

Recently, epigenetics research has confirmed that even if gene sequences do not change, epigenetic mechanisms via chromatin structure alteration and gene expression regulation are involved in most biological processes including organism development, cellular processes and survival. Thus, abnormal epigenetic regulation may be implicated in various diseases, such as cancers [1,2].

Histone post-translational modifications are critical for defining the global epigenetic status of a cell. For example, ethanol exposure has been shown to alter histone acetylation in the developing rat cerebellum, while in neural stem cells (NSCs) ethanol exposure reduced H3K27me3 and H3K4me3 at gene promoters involved in neural precursor cell identity and differentiation [3,4]. Also, increased histone H3 acetylation and decreased methyl CpG binding protein 2 (MeCP2) association with BDNF promoter IV were found in the medial prefrontal cortex of cocaine (a tropane alkaloid)-treated rats [5]. Moreover, increased gene silencing associated marker histone H3K27me3 has been observed in breast cancer MCF7 cells and mammary glands of six-week-old mice in the presence of bisphenol A (BPA), an endocrine disruptor which is widely used in plastic bottle manufacture [6]. In summary, perusal of literature indicates that altered epigenetic codes may affect organismal development or biological cellular process by misregulating gene transcription.

Arsenic contamination in drinking water has occurred in many countries including Bangladesh, India, China and Mexico, and more than 140 million people worldwide may be exposed to As concentrations exceeding the WHO’s (World Health Organization) safety standard of 10 μg/L [7]. Therefore, As-contamination has become a worldwide environmental concern. Although As compounds have been used as medicinal agents for centuries especially As_2_O_3_, which is effective for treating acute promyelocytic leukemia (APL), the clinical application of As_2_O_3_ is limited by its toxicity to the heart, liver, kidney, and nervous system [8,9]. Chronic exposure to As is also strongly associated with various types of tumor such as lung and bladder cancers [10,11]. Recently, increasing evidence suggests that arsenicals are suspected to affect biological responses by altering histone post-modifications. Exposure of cultured cells of *Drosophila* melanogaster to arsenite induces a severe deacetylation of core histones [12]. Also, global reduction of H3K9 acetylation occurred in peripheral mononuclear cells of subjects with exposure to As in their drinking water [13]. In addition, alteration of the histone modifications by environmental factors may be partly generated by directly regulating levels and/or activities of histone modifying enzymes [14]. For example, exposure to nickel, an environmental carcinogen, increased global histone H3K9 cell methylation via inhibiting histone demethylase JMJD1A [15].

The human ortholog of *yeast* Sas2 protein hMOF (or MYST1), forms at least two distinct multi-protein complexes-MSL and NSL, and is mainly responsible for histone H4K16 acetylation (H4K16ac) in mammalian cells [16,17]. Depletion of cellular hMOF leads to genomic instability, spontaneous chromosomal aberrations, cell cycle defects, reduced transcription of certain genes, defective DNA damage repair, and early embryonic lethality [18–21]. MOF depletion results in loss of acetylation in post-mitotic cells; loss of MOF results in loss of H4K16ac in purkinje cells, which results in back work walking [22] and loss of T-cell differentiation [23]. Interestingly, knockdown of hMOF in UROtsa cells not only reduced global H4K16ac, but it also induced sensitivity to As. In contrast, the global H4K16ac levels gradually decreased with increasing concentrations of As^lll^ in UROtsa cells [24]. However, relative histone H4K16ac progressively increased in As-exposed keratinocytes [25]. In either case, changes in H4K16ac in As-exposed cells are definite. In cells, the global status of histone H4K16ac is regulated by multiple enzymes. Except for hMOF, several enzymes such as SIRT1 deacetyltransferases are also involved in H4K16 acetylation [26]. Therefore, we must clarify which enzyme is responsible for misregulating global histone H4K16ac after As exposure.

To better understand imbalanced global histone H4K16 acetylation produced by As_2_O_3_ we studied HeLa or HEK293T cells, and we report that reduced global histone H4K16ac in As_2_O_3_-exposed cells is caused by loss of enzymatic activity of hMOF through As-hMOF direct binding. These data may provide novel perspectives for elucidating cellular mechanisms of As toxicity.

## Materials and Methods

### Antibodies and chemicals

Anti-H4K16ac (H9164) antibody was obtained from Sigma (St. Louis, MO). Anti-hMOF rabbit polyclonal antibody was obtained from Bethyl Laboratories (A300-992A, Montgomery, TX). Anti-HDAC4 (17449-1-AP) polyclonal antibodies were from Boster (China, Wuhan). Anti-tubulin (sc-58666) was acquired from Santa Cruz (Dallas, TX). Anti-GAPDH (NM_002046) rabbit polyclonal antibodies were raised against bacterially expressed proteins (Jilin University). Arsenic Trioxide (As_2_O_3_) was obtained from Beijing Xin Ding Pengfei Technology Development Co., Ltd. (CAS1327-53-3, China). p-Aminophenyl Arsenoxide (CAS1122-90-3, Cat No. A622500) was purchased commercially from Toronto Research Chemicals (TRC, Canada).

### Cell culture and As exposures

The present study was approved by the Ethics Committee of the School of Life Sciences of Jilin University. Human embryonic kidney (HEK) 293T (ATCC^®^ CRL-3216^™^) or HeLa (ATCC^®^ CCL-2) cells were used in this study. Cells were maintained in Dulbecco's modified Eagle's medium (Sigma) with 5% glucose and 10% fetal bovine serum. Then, 100 mg As_2_O_3_ was dissolved in 2.5 ml solution containing 10% NaOH, and the final volume of the solution was made up to 50 ml with PBS (final concentration: 2mg/ml). The pH of the final solution was adjusted to 7.2 with 1 M HCl and the solution was protected from light and stored at -20°C until use.

HeLa or HEK293T cells grown to 30% confluence were treated with As_2_O_3_ at different concentrations (0.2~0.8μM). Cells were harvested at 24, 48, or 72 hours according to the experimental design. Whole-cell extract (WCE) was prepared by adding 4 × SDS loading buffer, and total RNA was isolated using TRIzol^®^ LS Reagent (Invitrogen).

### Preparation of As-activated agarose and affinity purification

As-immobilized agarose was prepared according to previous reports with some modification [27]. Briefly, 50 μl NHS-activated agarose slurry was washed with coupling/wash buffer containing 0.1M sodium phosphate, 0.15M NaCl (pH 7.2), and the coupling reagent PAPAO (10 μmol) was added. The mixture was incubated at 4°C for 4 hours and the gel was washed sequentially with coupling/wash buffer. The remaining epoxy-active sites were blocked with 1 M ethanolamine (pH 7.4) in coupling buffer for 20 minutes at room temperature with gentle shaking. Finally, the gel was washed again with coupling/wash buffer, and the gel was kept at 4°C protected from light prior to use.

As-activated agarose was equilibrated with PBS to remove storage solution. Affinity purification was performed by mixing WCE and the binding mixture was allowed to react at 4°C overnight. The next day, the gel was washed with PBS twice, and the reaction was halted by adding 4×SDS loading buffer. Pulled down hMOF was quantified by Western blot with specific antibodies.

### Reverse transcription PCR (RT-PCR)

Total RNA (1 μg) from each sample was used as a template to produce cDNA with PrimeScript 1st Strand cDNA Synthesis Kit (Takara, Japan). hMOF and GAPDH mRNA was measured by quantitative real time PCR (qRT-PCR) with Eco^TM^ Real-Time PCR Syatem (Illumina, Gene Company Limited). All PCR reactions were finished under the following program: initial denaturation step was 95°C for 3 minutes, followed by 40 cycles of denaturation at 95°C for 30 seconds, annealing at 60°C for 30 seconds. The following qRT-PCR primer sets were used to measure hMOF and GAPDH mRNA level in As-exposed cells: hMOF, 5’-GGCTGGACGAGTGGGTAGACAA-3’ (forward) and 5’-TGGTGATCGCCTCATGCTCCTT-3’ (reverse), yielding a 227 bp products; GAPDH, 5’-ATCACTGCCACCCAGAAGAC-3’ (forward) and 5’-ATGAGGTCCACCACCCTGTT-3’ (reverse), yielding a 460 bp products.

### Luciferase reporter assay

HEK293T cells were cotransfected with 0.8 μg of pGL4 (Promega) which encodes firefly luciferase, 2 ng of the control plasmid *Renilla* luciferase vector (Promega) which encodes *Renilla* luciferase and effector plasmid expressing pGL4-hMOF using PEI reagent (Polysciences). Total effector plasmid in each transfection was adjusted to 0.8 μg with empty vector. After 48 hours, pGL4-hMOF transactivation activity was determined by measuring firefly and *Renilla* luciferase activities using the Dual-Luciferase Reporter assay kit (Promega) and by normalizing firefly to *Renilla* luciferase.

### HAT assay

Recombinant human histones and histone octamers were prepared essentially as previously described [28] and HAT assays were performed as described [29].

### Transient transfection or RNAi treatment

HeLa or 293T cells were cultured in 6-well tissue culture plates (~3×10^5^ cells/well) in DMEM medium (Sigma) containing 10% fetal bovine serum. Cells were transfected with 1–2 μg pcDNA/Flag-hMOF (pcDNA3.1 as control) to over-express hMOF and 40 pmol HDAC4 siRNA (Lot No. 2837) SMART pool (Jima, China) was used to knock down HDAC4. Then, 4 hours later, cells were cultured in medium containing different concentration of As_2_O_3_ (0.5–1.0μM). Then, 48 hours after transfection, WCE was prepared for Western blot.

### Immunofluorescent staining

Cells were cultured in 24-well plates containing a coverslips (8D1007, Nest) on each well. Cells were then exposed to As_2_O_3_ and 48 hours later, cells were washed in PBS, fixed with 4% paraformaldehyde (PFA) for 15 minutes at room temperature, permeabilized with 0.5% TritonX-100 in PBS for 5 min, followed by blocking with 1% BSA in PBS for 1 hour at 37°C. Then, cells were washed for 5 minutes in PBS-T three times, incubated with primary antibodies including hMOF (1:100 dilution), H4K16ac (1:400 dilution) or HDAC4 (1:400 dilution) at room temperature, then stained with FITC-conjugated secondary antibodies (1:300, Santa Cruz sc-2012). Cell nuclei were stained using Vectashield with DAPI (H-1200) (Vecter Libraries, Inc.). Fluorescent images were observed with Olympus BX40F Microscope (Olympus Corporation).

### ChIP assay

One 10 cm dishe (~1×10^7^) of 293T cells grown to ~80% confluence in the presence/absence of As_2_O_3_ in culture medium were used for each ChIP assay. ChIP assays were performed 2–3 times independently as described previously using hMOF antibody [30]. ChIP DNA was measured using quantitative real time PCR (qPCR) with Eco^TM^ Real-Time PCR System (Illumina, Gene Company Limited). Both ChIP and no-Ab signal were normalized to total input. Primer sets for qPCR on the promoter region of HDAC4 were as follows: HDAC4–1.5kb (-1504 to 1516bp), 5’-CAGCCAAGTCTCCAAAACCC-3’ (forward) and 5’-GGTCATTGCAAGAAATGCTTAGG-3’ (reverse); HDAC4–0.47kb (-476 to 538bp), 5’-CCGTGTTTACGGCCACTACTG-3’ (forward) and 5’-GCTGCTGGAGCTGCCAATT-3’ (reverse).

### Flow cytometry

HEK293T or HeLa cells were cultured in DMEM medium with or without As_2_O_3_ (1.6μM or 3.2 μM). 48 hours after transient transfection hMOF in 293T cells or siRNA knocked down hMOF in HeLa cells were harvested and stained with an Annexin V-FITC/PI Kit (KeyGEN Biotech, China). Cells were suspended with 500 μl binding buffer, then 5 μl PI and 5 μl Annexin V-FITC were added, and finally the cells were incubated at room temperature for 20 minutes in the dark, Apoptosis was assessed with flow cytometry (BD Accuri^™^ C6, BD Biosciences).

### MALDI-TOF mass spectrometry

Four peptide fragments of hMOF containing wild type C2CH zinc finger domain (residue 208 to 231aa, WLCEYCLKYMKYEKSYRFHLGQCQ), two mutated C2CH (C210/213A and C230A), and a peptide not containing C2HC (residue 247 to 270aa, SVYEVDGKDHKIYCQNLCLLAKLF) were synthesized by GL Biochem (Shanghai, China). The peptides were adjusted to a concentration of 100 μM with buffer containing 10 mM Tris-HCl (pH 8.0). The peptide solution was then incubated with As at room temperature for 1 hour in the ratio of 1:2 (peptide:As). Then, 1 μl of reaction mixture was added to an equal volume of a standard cyano-4 hydroxy-cinnamic acid matrix solution for spotting on a sample plate. The mass spectra of peptide with or without As was analyzed by MALDI-TOF-MS in a linear model using 5800 MALDI-TOF/TOF (AB Sciex).

### UV absorbance spectrometry assay

C2CH zinc finger peptides of hMOF were incubated with increasing amounts of As. UV absorption spectra were recorded (220-380nm) using a NanoDrop 2000 UV-Vis Spectrophotometer (Thermo).

## Results

### Reduction of global histone H4K16ac after As_2_O_3_ treatment

**Fig 1:**
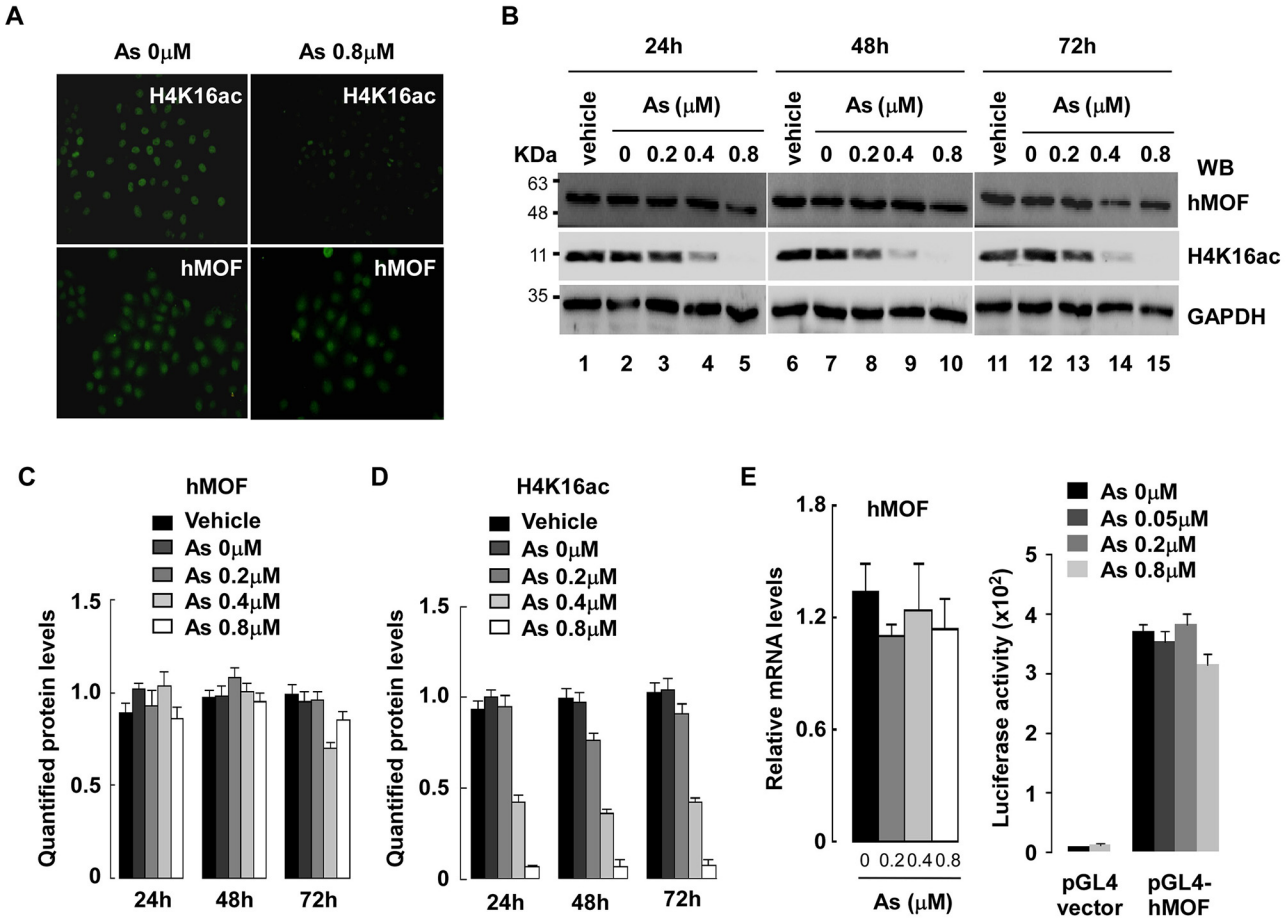
Decreased acetylation of histone H4K16 was observed in the presence of As_2_O_3_ HeLa or HEK293T cells. **(A)** Declined global H4K16ac in As_2_O_3_-exposed HeLa cells. Global acetylation of histone H4K16 and hMOF protein expression in As_2_O_3_-treated HeLa cells were measured with immunofluorescence and indicated antibodies. **(B)** A significant reduction of H4K16ac in As_2_O_3_-exposed 293T cells occurred. Representative results from three independent experiments are shown. hMOF and H4K16ac were measured with anti-hMOF and anti-H4K16ac antibodies and final protein signals were visualized with ChemiScope5000 (CLINX, China). **(C-D)** Quantified protein. Error bars represent standard error of means of 3 independent experiments. Blot images were scanned and signals were densitometrically quantified using Quantity One Basic software (Bio-Rad). Signals of hMOF and H4K16ac were normalized to GAPDH. **(E)** No changes of hMOF mRNA levels and hMOF transactivation in As_2_O_3_-exposed HEK293T cells. Cells were cultured in DMEM medium containing 0.2, 0.4 or 0.8 μM As_2_O_3_ for 48 hours. Relative mRNA levels of hMOF and GAPDH (as control) were measured with qRT-PCR (left panel). In addition, luciferase activity of pGL4-hMOF in As_2_O_3_-exposed 293T cells was measured and firefly values were normalized by renilla values (right panel).

As exposure is reported to induce global alteration of histone modification in human cells [12,13,24,25]. To clarify these effects on histone H4 specific lysine sites, we used immunofluorescent staining with acetylation-specific antibodies in As_2_O_3_-exposed HeLa cells, and we observed that H4K16ac was reduced (Fig 1A, upper panel), but other histone modifications of H4 did not change much (Figure A in S1 Text). Data were consistent with previous reports that showed histone H4K16ac is decreased after As treatment [24]. To understand whether histone H4K16ac reduction by As_2_O_3_ is due to reduced hMOF, we measured hMOF protein expression and observed that hMOF protein did not decline (Fig 1A, lower panel).

To confirm immunofluorescent data, we treated HEK239T cells for 24, 48 and 72 hours with As_2_O_3_. Then, WCE was quantified by Western blot with the indicated antibodies. At all times, As_2_O_3_ reduced global acetylation of histone H4K16 in a dose-dependent manner (Fig 1B). Hypo-acetylation of H4K16 occurred 24 hours after As_2_O_3_ exposure and hMOF protein expression did not change after As_2_O_3_ treatment at any time point (*p*>0.05), (see hMOF protein and global acetylation on histone H4K16 in Fig 1C and 1D). hMOF mRNA did not change as As increased (Fig 1E, left panel). To further clarify the correlation between As_2_O_3_-induced low histone H4K16ac and hMOF transactivation, luciferase activity of pGL-hMOF (containing hMOF promoter region from 1464 bp to +6 bp) was carried out in As_2_O_3_-exposed 293T cells (0.05–0.8 μM). The results as shown in Fig 1E (right panel) luciferase activity did not change at any As_2_O_3_ concentration.

### As_2_O_3_ directly binds to C2HC zinc finger domain of hMOF

**Fig 2:**
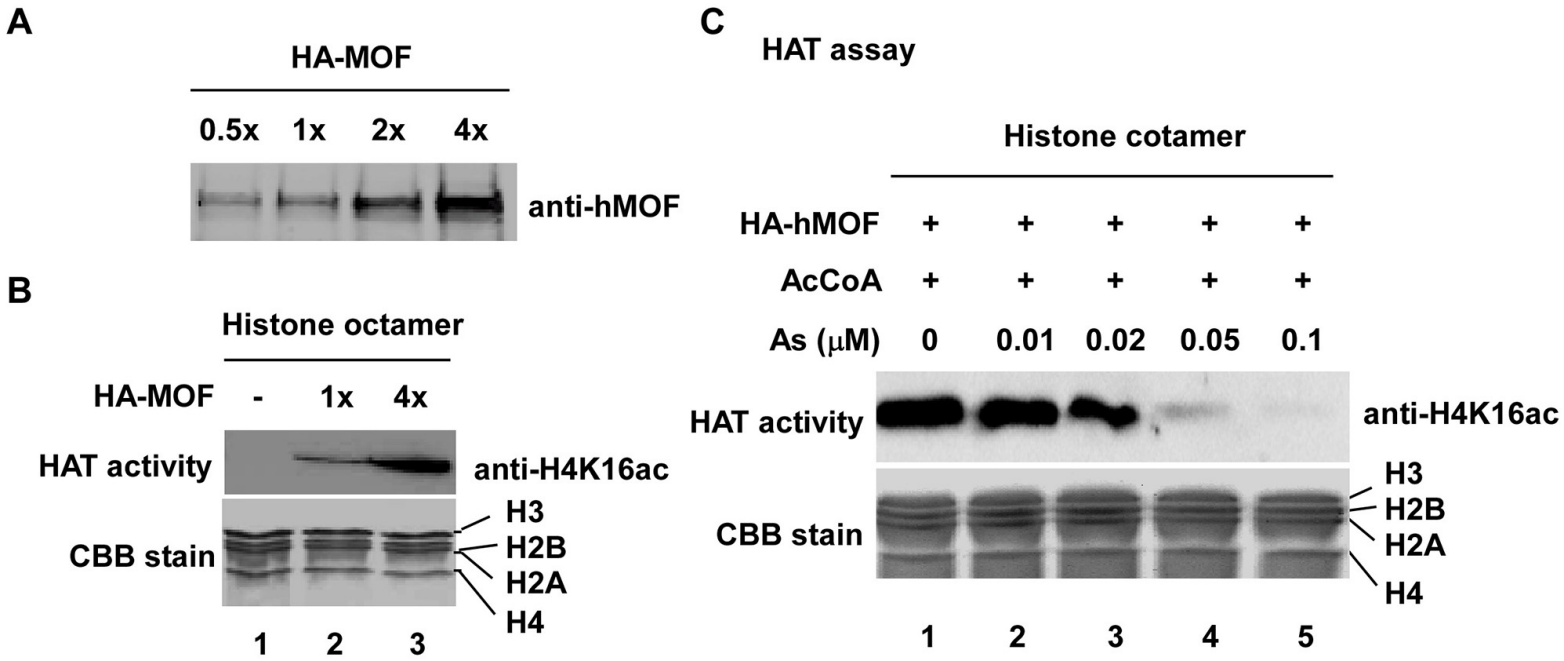
As_2_O_3_ inhibits hMOF HAT activity *in vitro*. **(A)** Recombinant hMOF. hMOF protein was measured with anti-hMOF antibody. **(B)** Insect cell expressed/purified HA-hMOF possessing HAT activity. Histones were visualized with Coomassie brilliant R250 blue (CBB) stain (middle panel) and H4K16ac was confirmed with acetylation-specific antibody (top panel). **(C)** Inhibitory effect of As_2_O_3_ on hMOF enzymatic activity.

**Fig 3:**
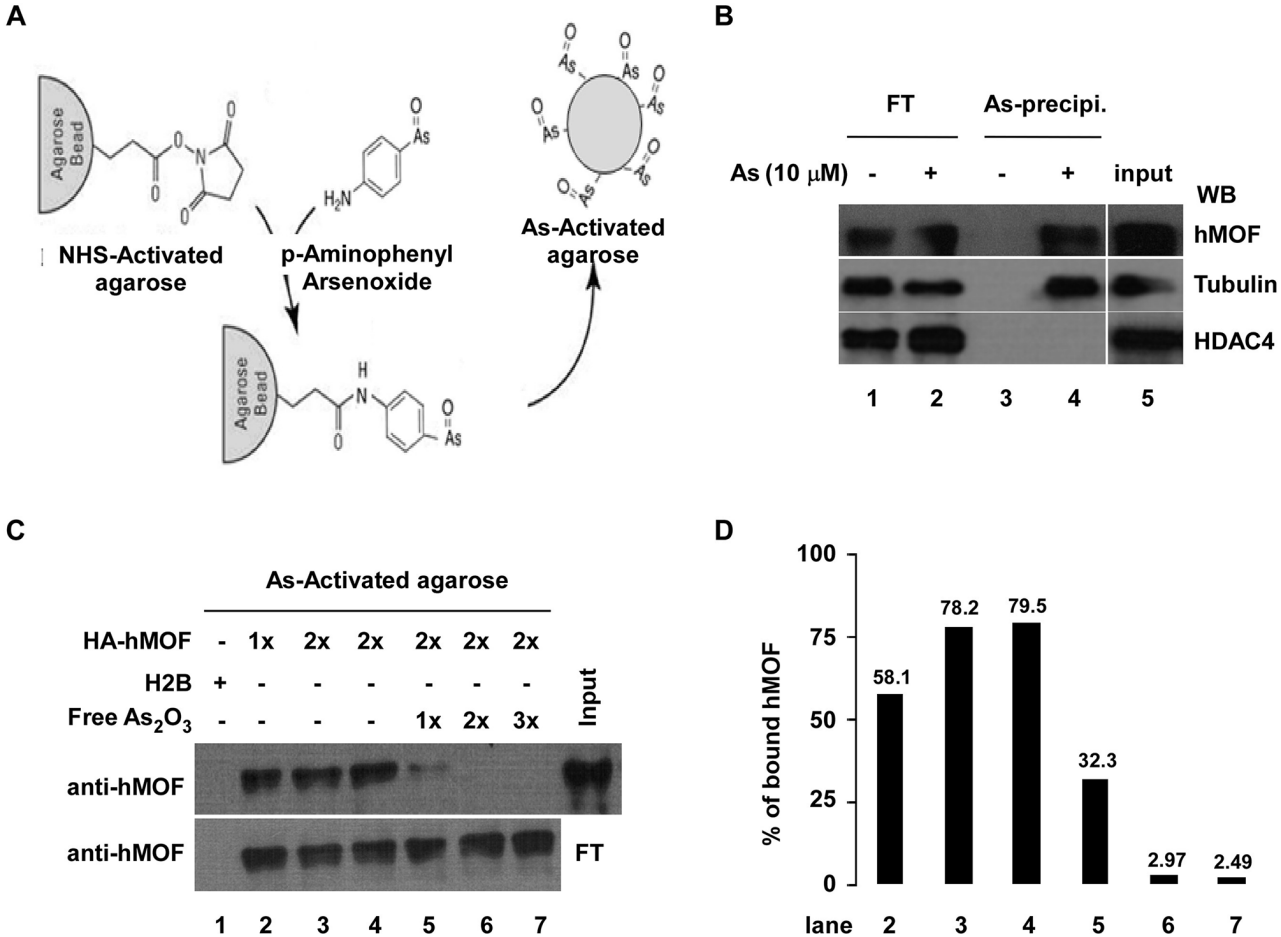
As_2_O_3_ can bind directly to hMOF. **(A)** Schematic of As-immobilized agarose preparation. **(B)** As-immobilized agarose pulled down hMOF. **(C&D)** Competitive inhibition between As and hMOF. H2B protein is as negative control. Western blot images were scanned and signals were densitometrically quantified with Quantity One Basic software (Bio-Rad). The percentage of bound hMOF in the flow through appears in **D**.

hMOF, as a catalytic subunit, forms two distinct cellular complexes-MSL and NSL in human cells [16,17]. Although the two complexes are composed of different proteins, both MSL and NSL possess acetylation activity on histone H4K16, suggesting that hMOF might be chiefly responsible for H4K16ac in cells [16,31]. Because As-induced decreases in H4K16ac may have caused hMOF activity loss, As was studied in the context of hMOF using an HAT assay. Insect cell expressed/purified hMOF (Fig 2A) with HAT activity (Fig 2B) was used to estimate the impact of As_2_O_3_ on hMOF activity. Data (Fig 2C) show that HAT activity of hMOF was blocked by As_2_O_3_ in a dose-dependent manner. As can inhibit several enzymes, such as GSH reductase [32] and lipoamide dehydrogenase [33]. Thus, As directly interact with hMOF protein. Using As-immobilized agarose (Fig 3A) we found that As-agarose pulled down hMOF protein (tubulin as positive control), but not HDAC4, indicating a physical interaction between As and hMOF (Fig 3B). These data were confirmed with competitive binding experiments with free As_2_O_3_ (10–100 μM). hMOF bound to As-agarose was gradually removed by increasing the amount of free As_2_O_3_ (Fig 3C and 3D).

**Fig 4:**
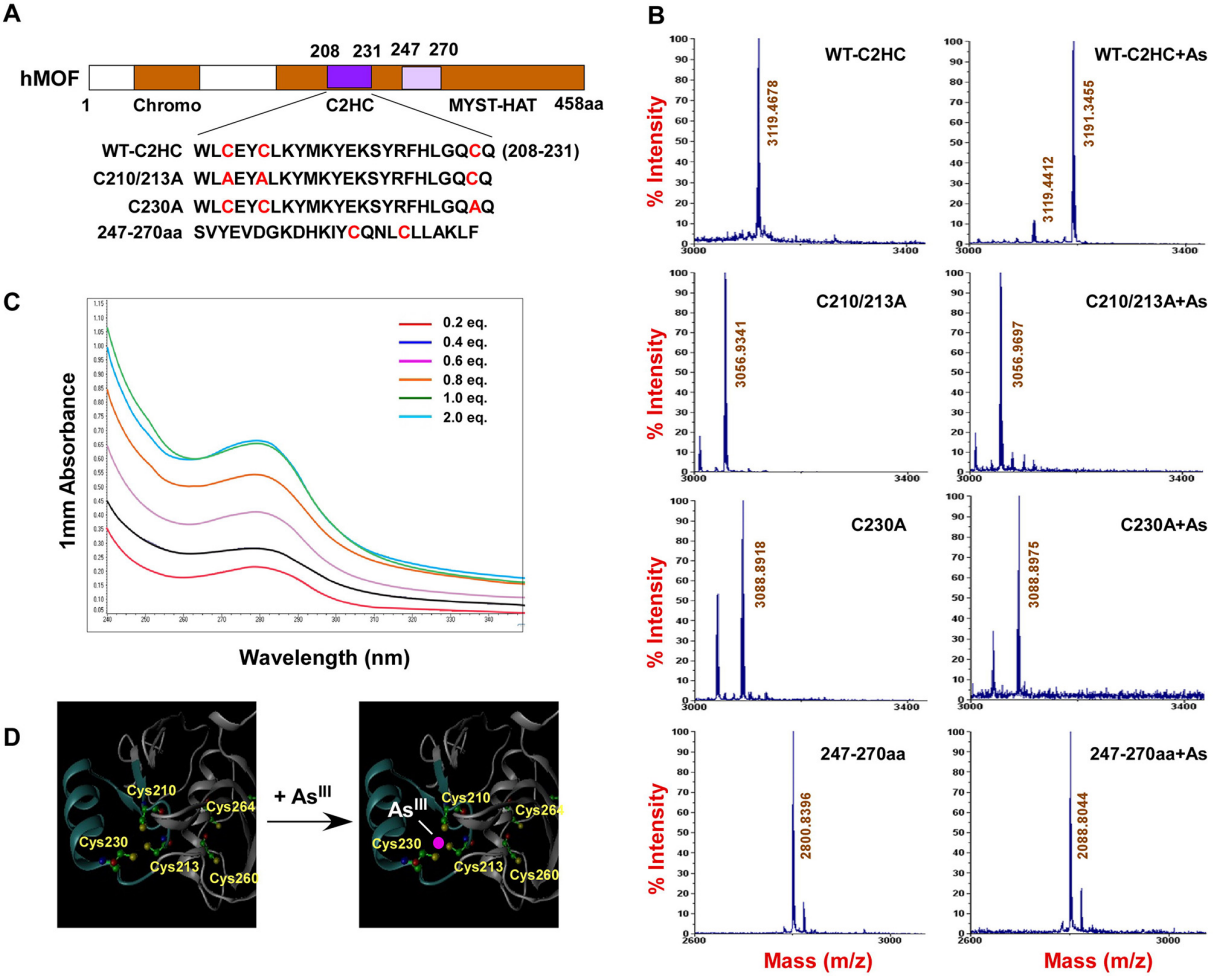
As_2_O_3_ binds directly to zinc finger (C_2_HC) peptide of hMOF. **(A)** Schematic of domain structure of hMOF. Chromo, chromatin organization modifier domain; C_2_HC, zinc finger domain; MYST-HAT, MYST-family histone acetyltransferase domain. The sequences from 208–231 and 247–270 residues are synthetic peptides of hMOF. **(B)** Analysis of MALDI-TOF mass spectra on interaction between As_2_O_3_ and synthetic peptides of hMOF. The molar ratio between the peptides and arsenic is 1:2. **(C)** UV absorption spectra of the zinc finger peptide. **(D)** Simulation of binding between zinc finger of hMOF and arsenic atoms. Three-dimensional structure based on the X-ray structure of hMOF (PDB code: 2GIV) [35]. All Cys residues on hMOF are represented in a ball-and-stick depiction. The zinc finger region-C2HC-type is dark cyan. Color coding: green, C; red, O; blue, N; yellow, S. The arsenic atom is pink.

It has been reported that As directly binds to the zinc finger domain of PML-RARα which is a fusion protein containing sequences from the PML zinc finger protein and retinoic acid receptor alpha [34]. hMOF also contains a conserved Cys2HisCys (C2HC) zinc finger domain. To investigate As binding to the C2HC zinc finger domain of hMOF to perturb histone H4K16ac, four peptides containing the wild type C2HC domain, a C210/213A mutated C2HC domain, a C230A mutated C2HC domain, and without a C2HC domain of hMOF were synthesized (Fig 4A). Four peptides were incubated with As at room temperature for 1 hour. Mass spectra of peptides with/without As were analyzed by MALDI-TOF-MS. Compared to MS spectra of synthetic peptides of hMOF, a +72 Da mass shift was only observed in the wild type C2HC domain of hMOF after incubating peptides with As, but not in the other three peptides (Fig 4B). Binding of As (III) to the C2HC zinc finger peptide released three protons, suggesting that As (III) bound to three Cys of wild type C2HC peptide. These data were confirmed by UV absorbance detection. Fig 4C shows that the optical absorbance curve of the wild type C2HC peptide of hMOF moved up with increasing amounts of As (250-340nm). According to the 3-D structure of hMOF (PDB code: 2GIV) [35], we speculated a potential binding mode between As and the C2CH zinc finger of hMOF (Fig 4D).

### hMOF attenuates cell death induced by As_2_O_3_

**Fig 5:**
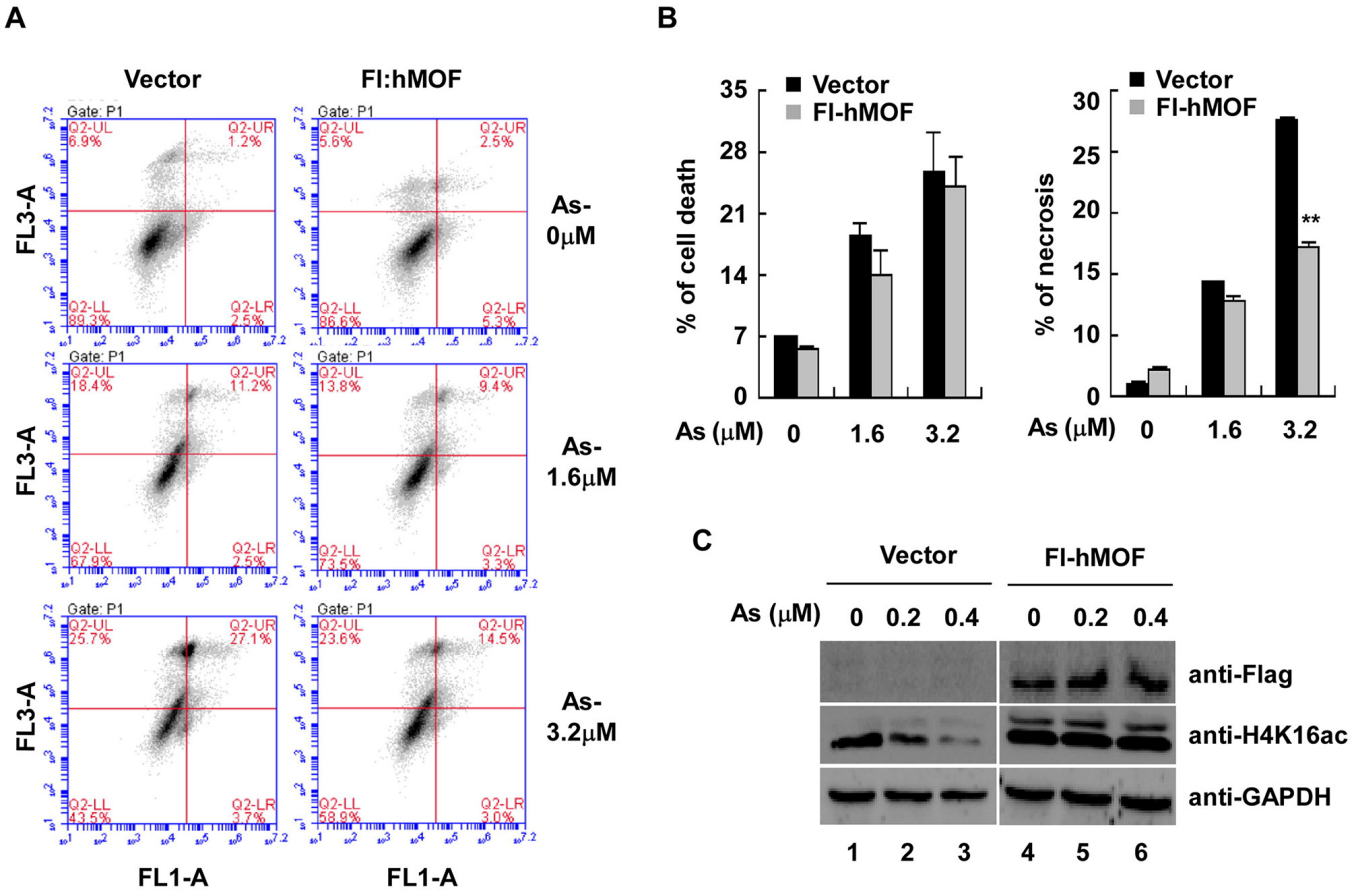
Over-expression of hMOF inhibited cell sensitivity to As_2_O_3_ in 293T cells. **(A)** Representative flow cytogram of Annexin V binding (X-axis) versus PI uptake (Y-axis) in 293T cells. Numbers in the upper left and right, lower left and right quadrants represent percentage of damaged, necrotic, live and apoptotic cells, respectively. **(B)** Quantified percentage of damaged and necrotic cells for **(A)**. Statistical significant difference expressed as **p<0.01 (Student *t*-test). **(C)** Reversion of declined H4K16ac in As_2_O_3_-exposed 293T cells. 48 h after As_2_O_3_ treatment (0.2 and 0.4 μM), cells were harvested and lysed. hMOF and global modification of H4K16ac were measured.

**Fig 6:**
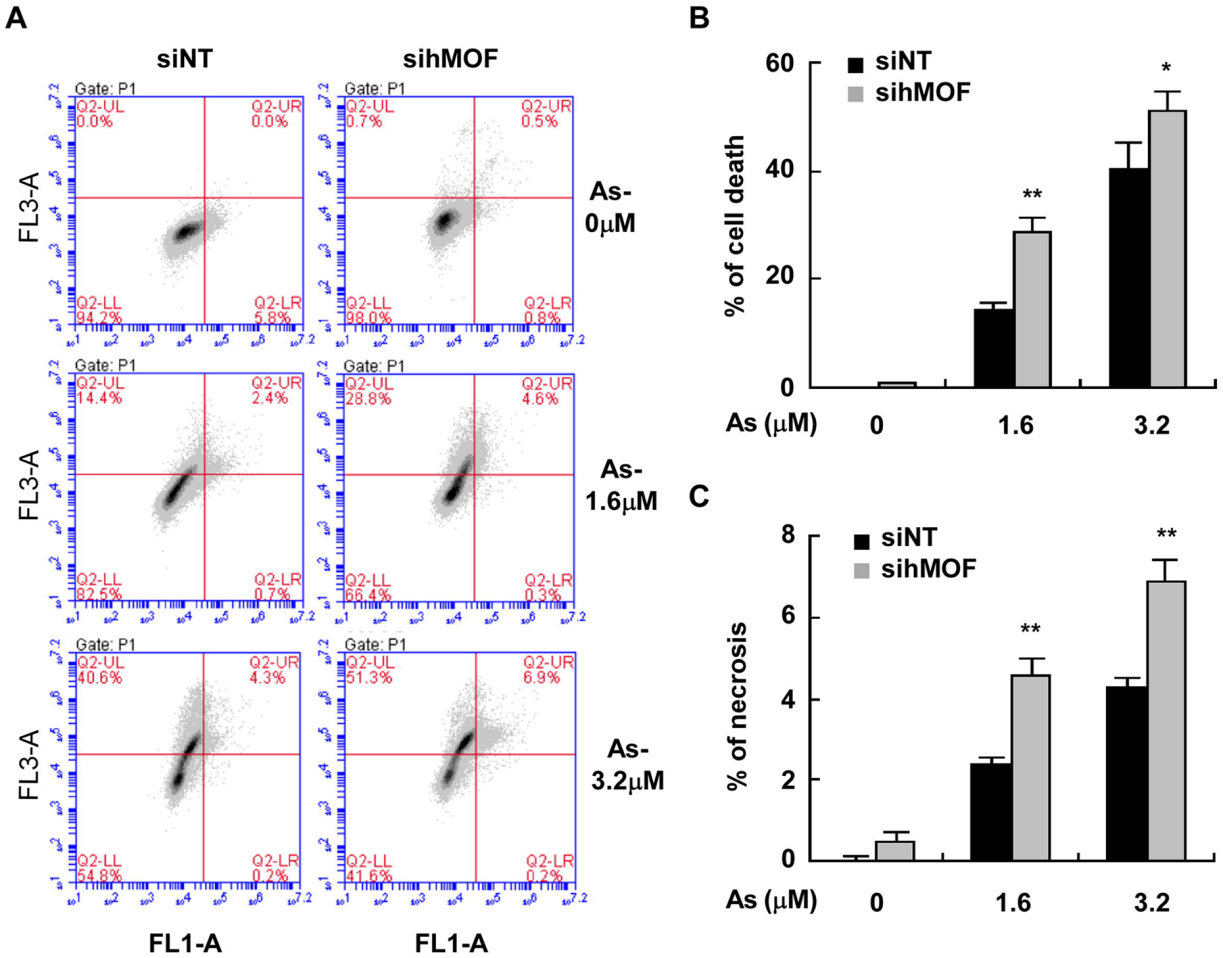
Knockdown hMOF promoted cell sensitivity to As_2_O_3_ in HeLa cells. **(A)** Representative flow cytogram of Annexin V binding (X-axis) versus PI uptake (Y-axis) in HeLa cells. **(B-C)** Quantified percentage of damaged and necrotic cells for (A). Statistical significant difference is expressed as *p<0.05 and **p<0.01 (Student *t*-test).

To confirm that As-produced global hypo-acetylation of H4K16 is due to reduced HAT activity of hMOF, we measured over-expression of hMOF with respect to cell sensitivity to As_2_O_3_. Flow cytometry of Annexin V binding/PI uptake was assessed in As_2_O_3_-exposed 293T cells (Fig 5A). Apoptosis is depicted in Fig 5B. Over expression of hMOF in As_2_O_3_-exposed 293T cells significantly inhibited necrosis at 3.2 μM As_2_O_3_ compared to vector only controls (*p<0.05). However, no statistical significant difference in damaged cells and apoptosis was observed between vector and hMOF ever-expressed groups (*p*>0.05). In addition, over-expression of hMOF blocked As_2_O_3_-induced global histone H4K16ac in 293T cells (Fig 5C). Therefore, over-expression of hMOF increased resistance to As_2_O_3_ and decreased toxicity in 293T cells. In contrast, knocking down hMOF in HeLa cells increased cell sensitivity to As2O3 (Fig 6A). Fig 6B and 6C show that knocking down hMOF in As_2_O_3_-exposed HeLa cells caused cell damage and death (**p<0.01 and *p<0.05, respectively) (**p<0.01 in both As_2_O_3_ groups) in As_2_O_3_-exposed HeLa cells.

### hMOF, but not HDAC4, caused modest global histone H4K16ac after As exposure

**Fig 7:**
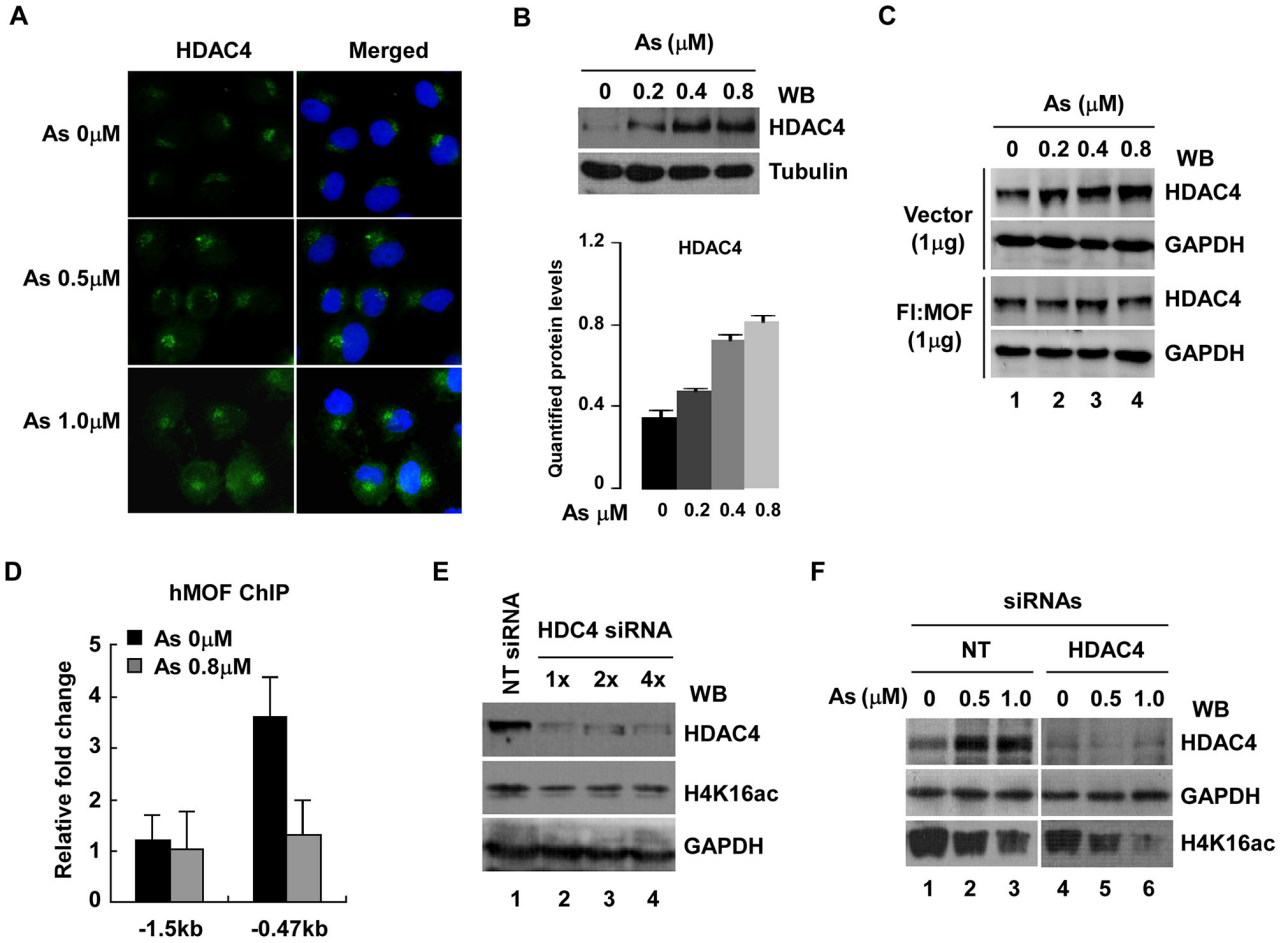
Reduction of global H4K16ac by As_2_O_3_ is due to the loss of enzymatic activity of hMOF, but not HDAC4. **(A)** As_2_O_3_-induced high expression of HDAC4 confirmed by immunofluorescence. **(B)** Increased HDAC4 protein by As_2_O_3_. **(C)** Overturn of the HDAC4 protein levels by over-expression of hMOF in As_2_O_3_-treatedt cells. WCE analyzed by Western blot with anti-HDAC4 antibody. **(D)** As_2_O_3_ blocked recruitment of hMOF on HDAC4 promoter region. ChIP assays were performed using hMOF specific antibody in As_2_O_3_ exposed 293T cells. ChIP DNA was measured by qRT-PCR with designed primer sets. **(E)** Effect of knockdown HDAC4 on global histone H4K16ac. **(F)** Knockdown of HDAC4 did not stop As_2_O_3_-induced reduction of H4K16ac. 3 h after HDAC4 siRNA transfection, cells were exposed to cell culture medium containing 0.5 and 1.0 μM As_2_O_3_, and 48 h later, cells were harvested. Proteins were measured with Western blot.

Histone acetylation, one of the best characterized epigenetic modifications, is controlled by histone HATs and histone deacetylases (HDACs). Recent studies suggest that the global modification status of H4K16ac is also affected by some HDAC complexes, such as SIRT1 and HDCA2 [26,36]. To know whether As_2_O_3_-induced decreases in histone H4K16ac was related with HDACs, we measured HDACs expression using Western blot and observed that As_2_O_3_-dependent increases of HDAC4 occurred. As shown in Fig 7A and 7B, elevation co-incident HDAC4 expression was confirmed and increased with increasing As concentration. Also, elevated HDAC4 was reversed by over-expressing hMOF (1 μg) (Fig 7C), providing evidence that hMOF may regulate cellular HDAC4 protein. To confirm this, ChIP assays were performed for hMOF in 293T cells with/without As_2_O_3-_treatment. Fig 7D shows that hMOF enriched at -0.47 kb upstream of the *HDAC4* transcriptional start site on HDAC4 was blocked in As_2_O_3_-exposed cells. To learn whether increased HDAC4 is involved in hypo-acetylation of histone H4K16, HDAC4 knockdown experiments were performed with specific siRNAs, but global histone H4K16ac did not increase after HDAC4 knock down (Fig 7E). Moreover, depletion of HDAC4 could not block reduction of histone H4K16ac in As_2_O_3_-exposed cells, suggesting that HDAC4 may not be directly involved in deacetylation of H4K16 (Fig 7F).

## Discussion

Perusal of literature reveals that post-translational modifications of histones are implicated in As-induced carcinogenicity. For instance, increased global histone H3K9me2 and decreased H3K27me3, both of which represent gene silencing markers, were observed in As-exposed cells [37]. Genome-wide analysis suggests that As exposure during embryonic development caused global hypo-acetylation at H3K9 [38]. In addition, Li’s group [39] reported that As-induced histone H3 phosphorylation might be responsible for the up-regulation of oncogenes *c-fos* and *c-jun*, suggesting that H3 phosphorylation may also contribute to As-induced carcinogenesis. Together, these studies suggest that As-mediated epigenetic mechanisms may be involved in its toxicity and carcinogenicity. Our data show that the reduction of global histone H4K16ac by As_2_O_3_ may be due to direct binding to histone acetyltransferase hMOF in cells.

Jo and coworkers reported that acetylated histone H4K16 by hMYST1 increased the resistance of UROtsa cells to As toxicity [24]. In our laboratory, we observed reduction of global histone H4K16ac in both As_2_O_3_-exposed HeLa or HEK293T cells (Western blot, immunofluorescence) and this reduction occurred after As exposure of 24–72 hours (Fig 1). Consistent with previous results, over-expression of hMOF not only increased global histone H4K16ac, but it also increased resistance of 293T cells to As. Transient transfection of hMOF in As_2_O_3_-exposed 293T cells significantly reduced necrotic cells as measured by flow cytometry of Annexin V /PI incorporation (Fig 5).

It has been reported that As exerts some of its biological effects through direct interaction to accessible thiols (SH) of cysteine residues (Cys) on protein, which are often located at active sites of many important enzymes, causing the thiol containing proteins/or enzymes to lose activity [40]. For example, by binding with Cys residues of estrogen receptors in MCF-7 breast cancer cells, As blocks the ligand-receptor reaction [41]. We confirmed a direct interaction between As and hMOF with As-immobilized agarose (Fig 3). Therefore, we suspect that physical interaction between As and hMOF might disturb histone H4K16 acetylation activity of hMOF. Consistent with this idea, the activity of hMOF HAT was directly inhibited by As_2_O_3_ in an *in vitro* HAT assay (Fig 2C). Reports suggest that As can bind directly to Cys residues in the zinc finger of PML-RARα [34]. hMOF also contains a C2HC zinc finger domain in which three Cys residues form a spatial triangle. To determine whether As also directly binds to this C2CH zinc finger, four sets of hMOF peptides were synthesized. Using MALDI-TOF mass spectrometry and UV absorbance detection approaches, we clarified the direct binding of As and the C2HC zinc finger peptide of hMOF (Fig 4). Hence, As may bind directly to SHs of three Cys residues on hMOF protein. These data and other reports suggest that histone H4K16ac contributes to cellular As resistance.

Interestingly, increased expression of HDAC4 protein was also observed in As_2_O_3_-exposed cells, and this elevated HDAC4 was inverted by over-expressing hMOF (Fig 7A, 7B and 7C), suggesting that hMOF may regulate cellular HDAC4 expression. As expected, a ChIP assay confirmed that hMOF was enriched at the -0.47 kb transcriptional start site of HDAC4 (-0.47 bp), however, this recruitment was obviously reduced in As_2_O_3_-exposed cells (Fig 7D). Moreover, decreased H4K16ac was not reversed by HDAC4 knockdown in As-exposed cells, in contrast, a slight decrease of global H4K16ac occurred after HDAC4 knockdown (Fig 7F). This may be explained by HDAC4 not being directly involved in de-acetylating histone H4K16. HDACs including HDAC4 have broader substrate specificity. Therefore HDAC4 knockdown had little effect for acetylation to a particular lysine site. However, it is important to note that the complex network systems exist for different enzymes, especially HATs and HDACs. Thus, a feedback pathway is initiated after depletion of HDAC4 [42]. Previous studies [43] reported that 3 μM As not only causes severe apoptosis, but also causes invasion of HSC5 cells, suggesting multiple toxicities of As. Although high expression of hMOF was detected in non-small cell lung cancer, low expression of hMOF and its corresponding H4K16ac were observed in many primary diagnosed cancer tissues including renal cell carcinoma, and breast and lung cancers [44–47]. For these cases, those tumor cells may be more sensitive to As_2_O_3_. Our experimental results verified this idea. Knocking down hMOF in As_2_O_3_-exposed human cervical carcinoma HeLa cells significantly increased damaged and necrotic cells (Fig 6), suggesting that hMOF promoted cell sensitivity to As. It has been reported that MOF is implicated in DNA damage repair [48]. Thus, increased damage repair capacity might be the possible mechanism for As resistance due to MOF and H4K16ac status. In contrast, raising global cellular H4K16ac through over-expression of hMOF in normal cells is protective against As toxicity (Fig 5). Thus, hMOF may be a target for treating As_2_O_3_ toxicity. Jens Füllgrabe and coworkers suggested that regulation of histone H4K16ac by hMOF can cause tumor cell autophagy during As_2_O_3_ treatment [49]. However, future studies are warranted to elucidate the precise interaction between As_2_O_3_ and histone H4K16ac.

## Conclusions

As a conclusion, As-induced global histone H4K16ac is partly due to the fact that As binds directly to the histone acetyltransferase hMOF protein and decreases HAT activity of hMOF. That hMOF is a direct target of As_2_O_3_ provides new perspectives for elucidating the toxicology of As on cells.

## Supporting Information

S1 Text (Figure A) Decreased acetylation of histone H4K16 was observed in the presence of As_2_O_3_ HeLa or HEK293T cells. (PDF)
