# Supplementary material for: Arsenic Trioxide Reduces Global Histone H4 Acetylation at Lysine 16 through Direct Binding to Histone Acetyltransferase hMOF in Human Cells
Source: PLoS One. 2015 Oct 16;10(10):e0141014. doi: 10.1371/journal.pone.0141014 (PMC4608833; doi:10.1371/journal.pone.0141014)
Supplement: S1 Text — (PDF) [file pone.0141014.s001.pdf]

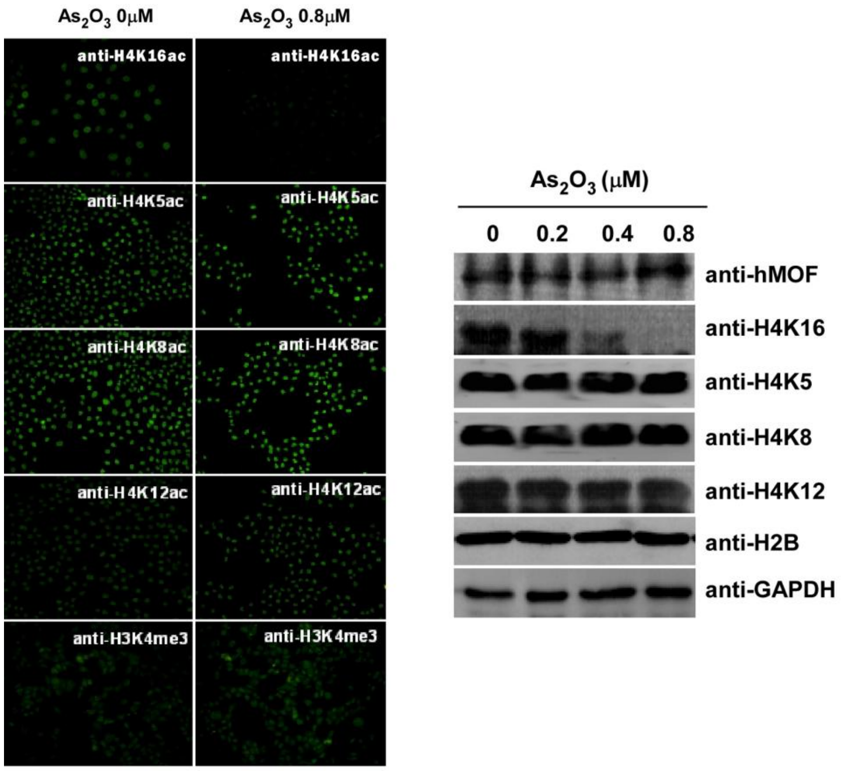

Figure A in S1 Text. **Decreased acetylation of histone H4K16 was observed in the presence of As_2O_3 HeLa or HEK293T cells. (Left panel)** Global modification levels of histone H4 at specific lysine site in As_2O_3-exposed HeLa cells. HeLa cells were cultured in 24-well cell culture plates in DMEM medium containing 10% fetal bovine serum. Cells were then grown to 30 % confluence, and treated with 0.8 μM As_2O_3 for 48 hours. Global modification on histone H4 or H3 and hMOF protein status in As_2O_3-presented HeLa cells were exhibited by immunofluorescence with indicated acetylation-specific antibodies**. (Right panel)** Significant reduction of histone H4K16ac levels in As_2O_3-exposed HEK293T cells. Cells were exposed to As_2O_3 in indicated concentration. 48 hours later, whole-cell extracts (WCE) were prepared and proteins were subjected to SDS-PAGE (12% or 18% gel). hMOF and acetylated specific residues on histone H4 were detected with indicated antibodies. GAPDH and H2B are as the internal control**.**
